# Fractional arm weakness as presentation of stroke due to posterior borderzone infarct: A report of two cases

**DOI:** 10.4103/0972-2327.74196

**Published:** 2010

**Authors:** Kaukab Maqbool Hassan

**Affiliations:** Department of Neurology, Command Hospital & Armed Forces Medical College, Pune

**Keywords:** Fractional weakness, borderzone cerebral infarction, parietal lobe, hand control

## Abstract

A 41-year-old male presented with acute onset weakness of the left hand. Magnetic resonance imaging (MRI) of the brain showed hyperacute infarct in the right middle cerebral artery (MCA)–posterior cerebral artery (PCA) watershed territory. Magnetic resonance angiography (MRA), Doppler ultrasonography, and digital subtraction angiography revealed severe right internal carotid artery (ICA) stenosis. The patient underwent carotid endarterectomy. The second patient was a 48-year-old male with acute onset right wrist drop. MRI of brain showed acute infarct in the left MCA–PCA watershed territory. MRA of brain and neck, Doppler ultrasonography of the neck vessels, and echocardiography were normal. Both the cases were not initially considered strokes by the referring physicians. Isolated hand palsy is a rare presentation of stroke, often mistaken for peripheral lesion. Fractional limb weakness as a presentation of acute ischemic stroke due to borderzone infarction involving parietal lobe is a rarely reported entity.

## Introduction

Isolated monoparesis caused by cerebral lesions is rare.[1,2] Isolated hand palsy is a rare presentation of ischemic stroke, often mistaken for peripheral lesion.[3,4] “Pseudoperipheral palsy” is a term used to describe a rare clinical feature consisting of predominant weakness of hand in association with cerebral infarction.[5] Discrete stroke in the parietal lobe or white matter of the angular gyrus, ventroposterior thalamus, and in the posterior limb of the internal capsule can mimic peripheral nerve lesions.[6]

The term “fractional arm weakness” has been applied when weakness of the hand differs from that of the shoulder.[3] Isolated hand weakness has been reported as a result of embolic stroke involving the hand knob area, large artery atherosclerotic infarct of vascular borderzones, and small subcortical lacunar infarct,[4] and rarely due to inferior parietal lobe infarctions due to severe carotid stenosis or dissection.[3,4]

## Case Reports

### Case 1

A 41-year-old male, chronic smoker (10 pack-years) and regular alcohol consumer, having no comorbidities, presented in May 2009 with weakness and numbness of the left hand on waking up. There was no other complaint. He was referred to Neurology Services on the fourth day of stroke. Power was Medical Research Council (MRC) grade 4 at left elbow flexor, grade 3 at left wrist, 50% left handgrip, and sensory impairment about 20% on both aspects of the left hand and volar forearm.

**Figure 1:**
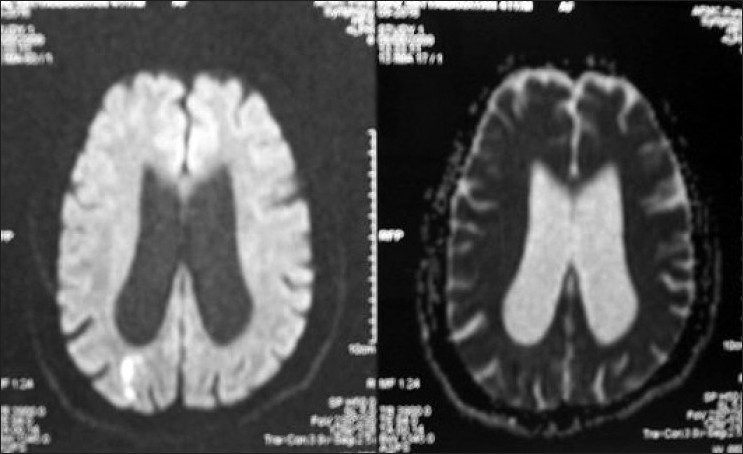
Diffusion-weighted image shows acute infarct in right parieto-occipital area

**Figure 2:**
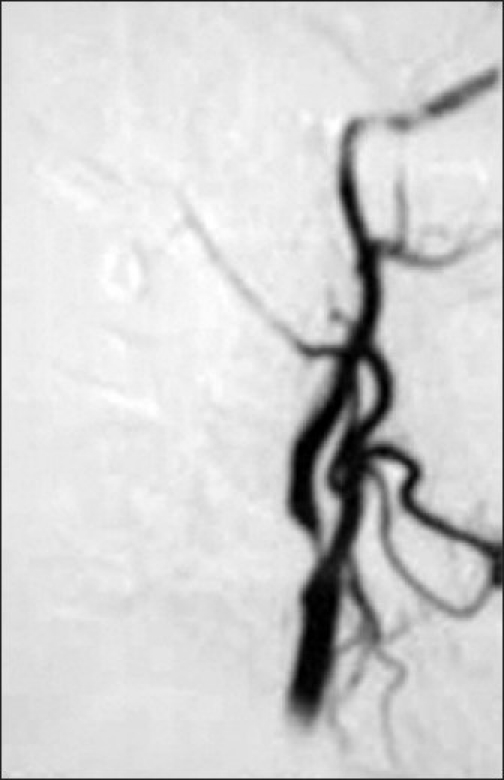
Digital subtraction angiogram shows severe stenosis at origin of right internal carotid artery

MRI of the brain showed hyperacute infarct in right MCA-PCA watershed territory [Figure 1]. MRA revealed >90% stenosis of proximal right ICA for 2.5 cm. Carotid Doppler ultrasonography showed 75% stenosis, whereas digital subtraction angiography showed >80% stenosis at origin of right ICA due to an eccentric plaque [Figure 2]. As the clinical findings were not conforming to nerve(s) distribution, nerve conduction studies were not carried out in this patient. Hemogram and metabolic profile were normal. Vasculitis and antiphospholipid workup was negative. Prothrombotic workup could not be done due to financial constraints.

The patient underwent right carotid endarterectomy. He had complete recovery from his weakness. Postoperative carotid Doppler study showed right CCA–peak systolic velocity (PSV) 63 cm/s and diastolic velocity 17 cm/s, right ICA–PSV 52 cm/s and diastolic velocity of 20 cm/s, with residual right ICA stenosis of 20% and uniform flow without turbulence or mosaic pattern. He is on aspirin and atorvastatin.

### Case 2

**Figure 3:**
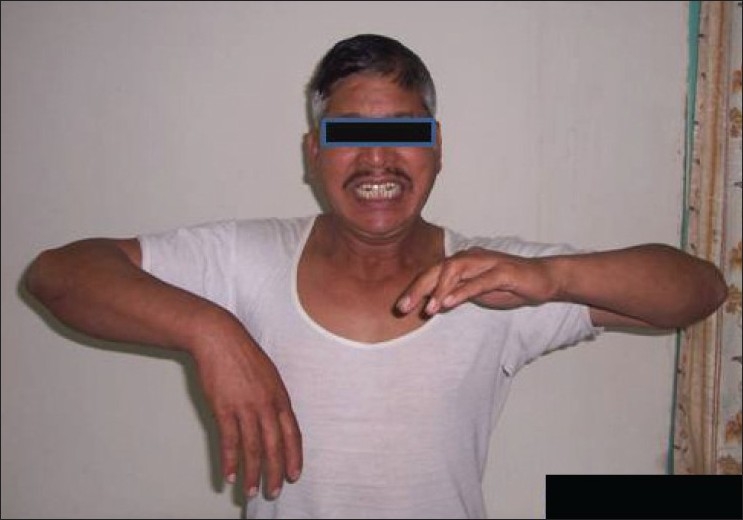
Second patient with right wrist drop

A 48-year-old male, chronic smoker (30 pack-years) and regular alcohol consumer, with no comorbidities, presented in August 2009 with right wrist drop [Figure 3] on waking up. There was no other complaint. He was initially worked up by the referring physician for radial nerve palsy, and referred to Neurology Services when nerve conduction study was normal. Examination revealed subtle facial asymmetry, power MRC grade 4 at right elbow flexor, grade 2 at right wrist extensor, grade 3 at wrist flexion, and 60% right handgrip. Sensory examination was normal.

**Figure 4:**
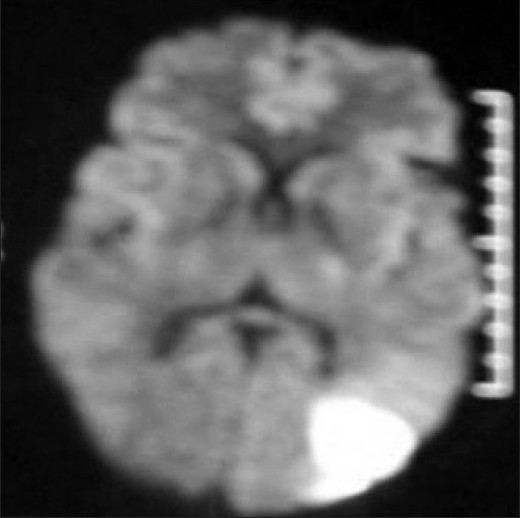
Diffusion-weighted image shows acute infarct in left parieto-occipital area

MRI of brain showed acute infarct in left MCA–PCA watershed territory [Figure 4]. The MRA of brain and neck was normal. Carotid Doppler ultrasonography and transthoracic and transesophageal echocardiography were normal. Hemogram and metabolic profile were normal. Vasculitis and antiphospholipid workup was negative. Prothrombotic workup could not be done due to financial constraints. He is on aspirin and atorvastatin.

## Discussion

Isolated hand palsies in association with cortical lesions have rarely been reported.[1] The frequency of isolated hand weakness due to acute ischemic stroke is unknown, but it is likely that many cases are initially mistaken for a peripheral lesion.[4] Two types of fractional weakness have been described, one predominantly involving the shoulder, the other the hand. Bilateral shoulder weakness referred to as “the man in the barrel,” occurs following cardiac arrest involving the MCA–ACA (anterior cerebral artery) borderzone. The term “pseudoperipheral palsy” has been used when weakness predominates in the hand.^3^

Celebisoy *et al*. reported eight patients presenting with isolated hand palsy due to discrete cortical infarction in the precentral gyrus.[5] It has been suggested that the motor hand area of the cerebral cortex is located either at the bottom of the central sulcus, upper portion of precentral gyrus, posterior bank of precentral gyrus, or precentral knob.[1] Functional MRI study has suggested that the ω- or ε-shaped area (the precentral knob) on the precentral gyrus in the axial plane is the cortical motor hand area.[1,6] An evolving isolated hand palsy has also been described as a result of infarction of the white matter of angular gyrus and without the involvement of the precentral knob.[3,6] Timsit *et al*. reported six patients who had evolving isolated hand palsy in the presence of symptomatic severe carotid stenosis and an infarction at the boundaries between anterior, middle, and posterior cerebral territory likely due to a decreased basal cerebral blood flow in the parietal lobe.[3] None of the patients had Babinski sign and all had mild sensory symptoms or signs in the affected hand.[3] All but one had an infarction of the angular gyrus, that is, posterior part of the inferior parietal lobule.[3]

Parietal lobe lesions are known to be associated with motor disorders in animal models as well as in humans.[2] It is possible that the inferior parietal lobe contains somatotopic representation of hand, and lesions here affect only motor aspect of perceptuomotor function of the hand.[3] These studies support the concept that widely spaced brain regions participate in the motor control of hand.[3,5,6]

Paciaroni *et al*. found that lesions were subcortical or in territory of posterior circulation in 32 of their 51 patients (62.7%) with isolated monoparesis studied over a period of nearly six years.[2] In their series, isolated monoparesis due to stroke was caused by small artery disease in about 40% of the cases.[2] Boundary zones were affected in three patients (parietal lobe) suggesting a hemodynamic infarct.[2] Recently, a patient with wrist drop has been reported following borderzone infarct due to spontaneous carotid artery dissection.[4]

The first patient had isolated hand weakness due to MCA–PCA borderzone hemodynamic infarction due to severe carotid stenosis. The mechanism of small and isolated cortical infarct reflects the likely small diameter of emboli, allowing them to be arrested in the smallest caliber arteries of distal MCA territory, in close proximity to anatomic border zone.[4] This was the likely mechanism of stroke in the second patient.

Consistent with the previous data, our cases suggest that the parietal lobe is involved in control of the motor function of the hand.[3] The major role of some parietal cortical regions is thought to be connected with motor organization and in particular with perceptuomotor coordination.[2] It is important to be aware of the broad differential diagnosis of isolated hand weakness and consider acute ischemic stroke as an uncommon underlying cause of these clinical presentations of hand weakness and wrist drop.
